# Testing bats in rehabilitation for SARS‐CoV‐2 before release into the wild

**DOI:** 10.1111/csp2.12707

**Published:** 2022-05-05

**Authors:** Scott Jones, Thomas Bell, Christopher M. Coleman, Danielle Harris, Guy Woodward, Lisa Worledge, Helen Roberts, Lorraine McElhinney, James Aegerter, Emma Ransome, Vincent Savolainen

**Affiliations:** ^1^ Department of Life Sciences, Georgina Mace Centre for the Living Planet Imperial College London London UK; ^2^ Queen's Medical Centre University of Nottingham Nottingham UK; ^3^ Bat Conservation Trust, Cloisters Business Centre London UK; ^4^ Department for Environment Food & Rural Affairs (Defra) London UK; ^5^ Animal and Plant Health Agency Surrey UK; ^6^ National Wildlife Management Centre Animal and Plant Health Agency York UK

**Keywords:** bats, coronavirus, COVID‐19, fecal RNA, quantitative PCR, rehabilitation, SARS‐CoV‐2, spillover

## Abstract

Several studies have suggested SARS‐CoV‐2 originated from a viral ancestor in bats, but whether transmission occurred directly or via an intermediary host to humans remains unknown. Concerns of spillover of SARS‐CoV‐2 into wild bat populations are hindering bat rehabilitation and conservation efforts in the United Kingdom and elsewhere. Current protocols state that animals cared for by individuals who have tested positive for SARS‐CoV‐2 cannot be released into the wild and must be isolated to reduce the risk of transmission to wild populations. Here, we propose a reverse transcription‐quantitative polymerase chain reaction (RT‐qPCR)‐based protocol for detection of SARS‐CoV‐2 in bats, using fecal sampling. Bats from the United Kingdom were tested following suspected exposure to SARS‐CoV‐2 and tested negative for the virus. With current UK and international legislation, the identification of SARS‐CoV‐2 infection in wild animals is becoming increasingly important, and protocols such as the one developed here will help improve understanding and mitigation of SARS‐CoV‐2 in the future.

## 1 ORIGIN OF SARS‐COV‐2 AND POTENTIAL FOR SPILLOVER

Since the start of the COVID‐19 pandemic, questions about the origin of the causative viral agent, severe acute respiratory syndrome coronavirus 2 (SARS‐CoV‐2; a sarbecovirus), continue to circulate (Burki, 2020). Multiple studies have suggested SARS‐CoV‐2 descended from a viral ancestor found in horseshoe (rhinolophid) and Old World leaf‐nosed (hipposiderid) bats (Luk et al., 2019; Platto et al., 2021; Shereen et al., 2020). This is perhaps unsurprising given that bats (Chiroptera) are the second most diverse order of mammals after rodents, with 1447 extant species (ASM, 2021) and host a wide diversity of viruses. Included in that number are viruses that have evolved to pose a risk to human health (Letko et al., 2020; Mollentze & Streicker, 2020), such as two other betacoronaviruses (Cui et al., 2018; Hu et al., 2015; Plowright et al., 2015): SARS‐CoV‐1 and Middle East respiratory syndrome coronavirus (MERS‐CoV). Further, viruses with high genetic similarity to SARS‐CoV‐2 have been sequenced from several horseshoe bat species (*Rhinolophus* spp.) in South‐East Asia, with some showing >96% genomic sequence identity with SARS‐CoV‐2 (Delaune et al., 2021; Murakami et al., 2020; Temmam et al., 2021; Wacharapluesadee et al., 2021; Zhou et al., 2020).

Despite the genetic similarity of these horseshoe bat betacoronaviruses to SARS‐CoV‐2, it has been suggested that bats were not the immediate source of infection of SARS‐CoV‐2, and that a bridging host facilitated viral evolution into humans (Mahdy et al., 2020; Platto et al., 2021). This echoes SARS‐CoV‐1 and MERS‐CoV, which passed to humans from bats via palm civets (*Paradoxurus* spp.) and camels (*Camelus dromedarius*), respectively (Dudas et al., 2018). Evidence suggests that SARS‐CoV‐2 can infect a multitude of both wild and domesticated animals, including domestic cats, captive lions, tigers, and other Felidae, ferrets, mink and otters, dogs, gorillas, white‐tailed deer, and many more (Halfmann et al., 2020; Hobbs & Reid, 2021; Kim et al., 2020; Sharun et al., 2021; Sit et al., 2020). This list is expected to continue to grow as more data emerges.

Recent research trying to understand the relationship between SARS‐CoV‐2 and bats indicate that susceptibility to infection varies with regard to the bat phylogeny. Although hipposiderid and rhinolophid species have shown a high prevalence for SARS‐related coronaviruses (SARSr‐CoV), evidence of transient SARS‐CoV‐2 infection has only been reported in the distantly related Egyptian fruit bat (*Rousettus aegyptiacus*; Schlottau et al., 2020). Hall et al. (2021) have described potential resistance to infection in another far more distant relative, the big brown bat (*Eptesicus fuscus*). Studies have reported instances of persistent and/or latent infections with other coronaviruses, in which the species of bats studied are asymptomatic despite confirmed infection (Baker et al., 2013). Further, MERS‐CoV replication in differing bat cell lines has also indicated the possibility of this virus‐producing persistent infection in several bat species (Banerjee et al., 2020; Caì et al., 2014). In instances of persistent infection in bats, it has been suggested that coronaviruses are shed episodically, which may provide an explanation for the variability of spillover events (Plowright et al., 2015).

To gauge the potential threat of transmission from bats to humans, a recent study of SARS‐CoV‐2‐like sarbecoviruses in South and South‐East Asia estimated around 400,000 people could be infected with such viruses annually in these regions, as a result of spillover events that do not produce disease or propagate into detectable outbreaks (Sánchez et al., 2021). Further, during the initial outbreak, the identification of two apparent lineages of SARS‐CoV‐2 (A and B) has led to the hypothesis that the current SARS‐CoV‐2 outbreak may be the result of two or possibly more spillover events from intermediary species (Mallapaty, 2021). Assuming that viral spillover is bidirectional, and that anything a wild animal might give us may also be passed from humans into wild animals, we identify a potential tension between humans and wildlife, as every possible host species needs to be treated with caution, an issue which is especially problematic for those working in the animal husbandry and biodiversity conservation sectors. To allow animal rehabilitation and conservation work to continue unabated, as well as to minimize the risk of human‐to‐bat transmission, we need rapid, accurate, and practicable protocols for identifying SARS‐CoV‐2 infections in wild animals. Many bats come into close contact with humans through shared habitat use (e.g., roosting sites in buildings) and more directly through handling of individuals in bat conservation programs both in the United Kingdom and more widely. Here, we propose a reverse transcription‐quantitative polymerase chain reaction (RT‐qPCR)‐based protocol for detection of SARS‐CoV‐2 in bats that have been in close proximity to humans using fecal sampling.

## 2 BATS AND POLICIES IN THE UNITED KINGDOM

Of the more than 1400 species of bats globally, only 17 are known to breed in the United Kingdom, and all are protected under the Wildlife Countryside Act of 1981 and the Conservation of Habitats and Species Regulations 2017 (UK Government, 1981, 2017). Organizations involved in the monitoring, conservation, and protection of bats, such as the Bat Conservation Trust, ecological consultants, and other organizations, routinely carry out population surveys, field‐based research projects, educational outreach events, and bat rescue. Many of these activities involve close proximity or even direct contact between humans and bats, which raises the potential risk for spillover of SARS‐CoV‐2 from humans to bats. Common et al. (2021) investigated this risk with regard to human‐to‐bat transmission during conservation fieldwork, concluding that the threat was low when following risk management protocols, such as the use of personal protective equipment (disposable gloves and face coverings) and other biosecurity measures. However, their assessment addressed a fieldwork‐based setting in open areas, where human–bat interactions are limited. The risk of spillover could increase when bats are housed in captivity as part of rehabilitation efforts. Here, we focus specifically on the latter scenario, and the testing of bats for SARS‐CoV‐2 prior to their return to the wild.

Further, several organizations also provide advice and support to the public through schemes such as the National Bat Helpline in the UK, run by the Bat Conservation Trust. In 2020 alone, the helpline received over 13,500 enquiries, of which over 6000 were regarding care of bats (Bat Conservation Trust, 2020). As part of this helpline, over 250 registered persons are available to aid in the rehabilitation of injured bats for release back into the wild. Registered bat rehabilitators have received relevant training to aide in this role, including bat health assessment and identification, public engagement, risk assessment, and legal requirements, approved by experienced trainers. Unlike other activities which involve handling bats, a license is not required for care and rehabilitation purposes in the United Kingdom, except where bats are to be kept in captivity for 6 months or more. This means that, in the United Kingdom, opportunities for spillover are not limited to registered rehabilitators and can include those in the general public that contribute to these limited (<6 months) rehabilitation efforts. Legislation regarding the handling and housing of bats will of course differ between countries. Overall, therefore, bats in rehabilitation, as even cats or dogs, may represent a pool of potential SARS‐CoV‐2 hosts. In addition, the constant changes in SARS‐CoV‐2 case numbers make it hard to estimate the risk at which rehabilitated bats are being put.

In October 2021, in England alone, over 1,000,000 people tested positive for SARS‐CoV‐2, with nearly triple that number identified as coming into close contact by NHS Test and Trace (UK Government, 2021a). Overall, 1:14 people were either confirmed positive or at risk of SARS‐CoV‐2 infection. It is therefore likely that, over the course of the pandemic, a number of bat rehabilitators may have contracted SARS‐CoV‐2 or been in close contact with an infected person.

Currently, guidelines in place to mitigate the risk of human‐to‐animal transmission of SARS‐CoV‐2 include those published by the World Organisation for Animal Health (2020 “Guidelines for Working with Free‐Ranging Wild Mammals in the Era of the COVID‐19 Pandemic”) and the Bat Specialist Group of the International Union for Conservation of Nature's Species Survival Commission (2021 “Recommendations to reduce the risk of transmission of SARS‐CoV‐2 from humans to bats in bat rescue and rehabilitation centers”) (IUCN BSG, 2021; World Organisation for Animal Health, 2020). In these guidelines, a risk management strategy of “Minimise, Assess and Protect” (MAP) is followed to design safe practices. As such, initially, the risk posed by or to an animal is determined by assessing if direct contact is indeed required or can be postponed. Where a risk is identified, the ability to minimize it through use of alternative or adapted practices is established, such as collection of fecal samples to replace oral/rectal swabs, as the former do not require direct animal contact. In addition, it is suggested that the three R's (“Replace, Reduce, and Refine”) principle for ethical use of animals in scientific research be considered at this step. Finally, risk can be further reduced through use of protective measures such as using personal protective equipment and disposable/disinfected equipment for each animal to reduce the risk of human‐to‐animal or animal‐to‐animal transmission.

As part of these guidelines, it is recommended that rehabilitators who have tested positive for SARS‐CoV‐2 isolate animals in their care pending further testing before release back into the wild (IUCN BSG, 2021; World Organisation for Animal Health, 2020). In doing so, the potential exposure of SARS‐CoV‐2 to wild bat populations is prevented and time allowed for detection of potential SARS‐CoV‐2 infection in‐housed wildlife. The latter is of particular importance during the current pandemic as under UK legislation (“The Zoonoses Order”) and as part of our membership of the World Organization for Animal Health, the reporting of SARS‐CoV‐2 infection in all wild and domesticated animals is a mandatory requirement (UK Government, 2021b). Further to this, bats identified to be in the vicinity of rehabilitators suspected SARS‐CoV‐2 positive or a known contact of such individuals also require isolation and testing prior to release back into the wild.

## 3 PROTOCOL TO TEST BATS FOR SARS‐COV‐2 AND RELATED CORONAVIRUSES

To address the need for SARS‐CoV‐2 diagnostic testing of bats following suspected exposure, an RT‐qPCR based protocol was developed, in which two gene regions of the SARS‐CoV‐2 genome were targeted for detection, the envelope (E) and nucleocapsid (N1) genes (Corman et al., 2020; Lu et al., 2020). Bats testing negative through this protocol for both genes would be considered suitable for release back into the wild following available guidance.

For determination of sampling methodology, human diagnostic strategies were considered. In humans, testing has focused on detection of SARS‐CoV‐2 in oropharyngeal/nasal swabs in conjunction with qPCR or lateral flow‐based methods. Obtaining analogous samples from bats would require collection to be carried out by trained and licensed rehabilitators, with collection of sufficient material for reliable detection requiring prolonged handling, with increased risk of viral spillover and causing undue stress and harm to animals. Another diagnostic sample is blood, but in the United Kingdom, this would require further training (MRCVS or Animals (Scientific Procedures) Act 1986 (ASPA) approved worker). In contrast, fecal samples are more accessible, require limited handling (if any) and can be collected with limited training. Because this approach is noninvasive, usually only local ethical approval may be required. Indeed, SARS‐CoV‐2 can be successfully detected in fecal samples from infected humans. Further, fecal sampling for detection of coronaviruses in bats has been used for monitoring coronaviruses, including SARS‐CoV‐1 (Berto et al., 2018; Kim et al., 2016; Lo et al., 2020; Ruiz‐Aravena et al., 2021; Wu et al., 2020).

In the present protocol, feces from bats potentially exposed to SARs‐CoV‐2 are to be collected at three time points at 5‐day intervals (Days 1, 6, and 11). Sample numbers and time period were selected based on a previous study on MERS‐CoV (Munster et al., 2016). Munster et al. (2016) reported shedding in experimentally infected Jamaican fruit bat (*Artibeus jamaicensis*), detectable by RT‐qPCR between 1‐ and 9‐day postinfection, when testing rectal swabs. As variable viral shedding was observed in this initial period and previous data have suggested episodic viral shedding in bats (Plowright et al., 2015), the collection of multiple fecal swabs increases the reliability of results.

For sample collection, a kit is provided by Imperial College London to rehabilitators housing and handling bats. This kit contains personal protective equipment (disposable nitrile gloves and face covering), sampling equipment, and a step‐by‐step sampling protocol, including biosafety recommendations. As two species of bats are known hosts for lyssaviruses in the United Kingdom, the handling of bats should be avoided if possible (Van der Jeucht et al., 2021). If handling is necessary, appropriate gloves (e.g., nitrile, leather, etc.), face covering, and possibly eye protection need to be worn. In addition, in the field, long sleeves and long trousers are recommended to limit skin exposure. These measures not only protect humans but bats too. Risk of transmission of SARS‐CoV‐2 by respiratory routes can also be reduced by increased ventilation of the room where the animal is kept. It is also recommended to isolate bats being rehabilitated from other animals.

Three 10 ml cryotubes, each containing 5 ml of RNAlater solution, are also provided in the kit. RNAlater stabilizes RNA, preventing degradation during collection, transport, and subsequent laboratory processing or archiving. For sample collection, we recommend that 0.02–1 g of feces is collected per time point, which is then to be returned to the laboratory for analysis. We recommend the collection of fresh feces without handling the bat. Once tested, results are to be returned to rehabilitators within 7 days, with positive results being directly reported to the Animal and Plant Agency (APHA) in the United Kingdom by Imperial College London. Half of the original samples are stored at −20°C and provided to APHA following positive results for secondary ISO 17025 accredited confirmatory testing. As SARS‐CoV‐2 is now a mandatory reportable infection in animals in England, Scotland, or Wales under the Zoonoses Order (As amended in 2021), all positive results from any animal must be promptly reported to the APHA (UK Government, 2021b).

For testing of samples, viral RNA is extracted from feces using the QIAamp Viral RNA Mini Kit (Qiagen), following the manufacturer's protocol for viral RNA extraction from stool, with the following amendments: up to 0.5 g of feces was added to 2 ml of 0.9% sodium chloride solution and vortexed. The mixture is then centrifuged at 6000 rpm for 2 min and the supernatant is filtered through a 0.22‐μM filter to remove fecal debris. Of this supernatant, 280 μl is carried forward for RNA extraction with RNA eluted in a final volume of 80 μl AVE Buffer and stored at −20°C. Successful RNA extraction is confirmed by quantification by Qubit 2.0 fluorometer (Invitrogen).

Further, to ensure RNA extraction is successful and produces PCR‐quality RNA, an RT‐qPCR assay targeting two genes within the bat genome, 18s rRNA and GAPDH, is carried out (Cowled et al., 2012; Wu et al., 2013). This is done using the Superscript III Platinum SYBR Green One‐Step RT‐qPCR Kit (Invitrogen). About 10 μl reaction mixtures consist of 1× SYBR Green Reaction Mix, 0.2 μl SuperScript III RT/Platinum Taq Mix, 1 μl of template RNA (0.4–3 ng/μl), 0.2 μM forward primer, and 0.2 μM reverse primer (primer sequences are provided in Table 1). Duplicated reactions are amplified using a Lightcycler 480 II starting at 50°C for 3 min (reverse transcription), then 95°C for 15 min (initial denaturation), followed by 45 cycles of 90°C for 15 s (denaturation) and 60°C for 45 sec (annealing and extension). A melt curve is generated for product analysis. Samples that show successful amplification are suitable for testing for SARS‐CoV‐2. In cases of negative results, extractions should be repeated.

**TABLE 1:** Primer and probe sequences for qPCR assays for amplification of SARS‐CoV‐2 and bat genes

Targeted gene	Name	Sequences (5′ – 3′)	References
SARS‐CoV‐2 E gene	E_Sarbeco_Fwd E_Sarbeco_Rev E_Sarbeco_Probe	ACAGGTACGTTAATAGTTAATAGCGT ATATTGCAGCAGTACGCACACA [6FAM]‐ACACTAGCCATCCTTACTGCGCTTCG‐[BHQ1]	Corman et al. (2020)
SARS‐ CoV‐2 N1 gene	N1_Fwd N1_Rev N1_Probe	GACCCCAAAATCAGCGAAAT TCTGGTTACTGCCAGTTGAATCTG [6FAM]‐ACCCCGCATTACGTTTGGTGGACC[BHQ1]	Lu et al. (2020)
18s rRNA	18s rRNA F 18s rRNA R	CACGGCGACTACCATCGAA CGGCGACGACCCATTC	Cowled et al. (2012)
GAPDH	GAPDH 1F GAPDH 1R	TGACCCCTTCATTGACCTCAAC TGACCGTGCCTTTGAACTTG	Wu et al. (2013)

For our SARS‐CoV‐2 RT‐qPCR reaction, the Lightcycler 480 RNA Master Hydrolysis Probes Kit is used. The reaction mixture (10 μl) for both genes (E and N1) consist of 1× Lightcycler RNA Master Hydrolysis Probe solution, 3.25 mM manganese (II) acetate solution, 1× Enhancer solution, 0.25 μM probe, 1 μM forward primer, and 1 μM reverse primer. For testing, 1 μl of template RNA (0.4–3 ng/μl) is used in duplicate. Reaction conditions using the Lightcycler 480 II machine are set at 63°C for 3 min (reverse transcription), then 95°C for 30 s (initial denaturation), followed by 45 cycles of 95°C for 15 s (denaturation), 60°C for 30 s (annealing) and 72°C for 1 s (extension).

Finally, the suitability of our primer sets (Table 1) for the detection of SARS‐CoV‐2 variants was assessed by aligning our primer and probe sequences against reference genomes of the Alpha, Beta, Gamma, Delta, and Omicron as well as the original Wuhan‐1 variant (Table 2). There was a 100% match for all sequences except 1 mismatch between the Omicron variant and position 2 from the 5′ end of primer “E_Sarbeco_Fwd” and probe “N1_probe” (Table 1). However, it is unlikely these single mismatches would prevent the detection of the Omicron variant as DNA polymerase extension is from the 3′ end of the primer.

**TABLE 2:** Genomes of SARS‐CoV‐2 variants used for the validation of our primer and probe sets

SARS‐CoV‐2 variant	Country of origin	GenBank/EBI accession number
Alpha	Bangladesh	MW624725
	Thailand	MZ888515
	UK	MZ344997
Beta	Germany	MZ433432
	Ghana	MW598419
	UK	OD927817
Gamma	Brazil	MZ169911
	Switzerland	OU267843
	USA	MW963223
Delta	India	MZ359841
	Morocco	MZ208926
	Thailand	MZ888532
Omicron	Bangladesh	OM570259
	Belgium	OL672836
	South Africa	OM739181
Wuhan‐1	China	MN908947

For quantification, the 2019‐nCoV positive control plasmids consisting of a pMB1 backbone with an E or N1 gene insert derived from Wuhan‐1 SARS‐CoV‐2 isolate (Accession no. NC_045512.2) are used (Integrated DNA Technologies). Plasmids are serially diluted at 1:10 and run at known concentrations (100,000 copies to 10 copies) alongside every reaction. Using the derived *C*_*q*_ values, a standard curve is generated for quantification of positive results and the limits of detection and quantification (LOD and LOQ, respectively) are calculated. Using these plasmids, the LOD and LOQ for detection of SARS‐CoV‐2 were 10 and 100 copies for the N1 gene, and 100 and 1000 copies for the E gene, respectively. Outputs from RT‐qPCR reactions are analyzed using the Lightcycler 480 Software. A sample is positive when both E and N1 genes amplify by RT‐qPCR; if only one of the two genes amplifies, it may indicate that the sample was positive for another coronavirus, but not SARS‐CoV‐2. For release, in line with available guidelines, bats must test negative for both genes (IUCN BSG, 2021; World Organisation for Animal Health, 2020).

These protocols have been used routinely in our laboratory at Imperial College London and successfully detected SARSr‐CoV (but not SARS‐CoV‐2) in bat fecal samples (unpubl.).

**FIGURE 1:**
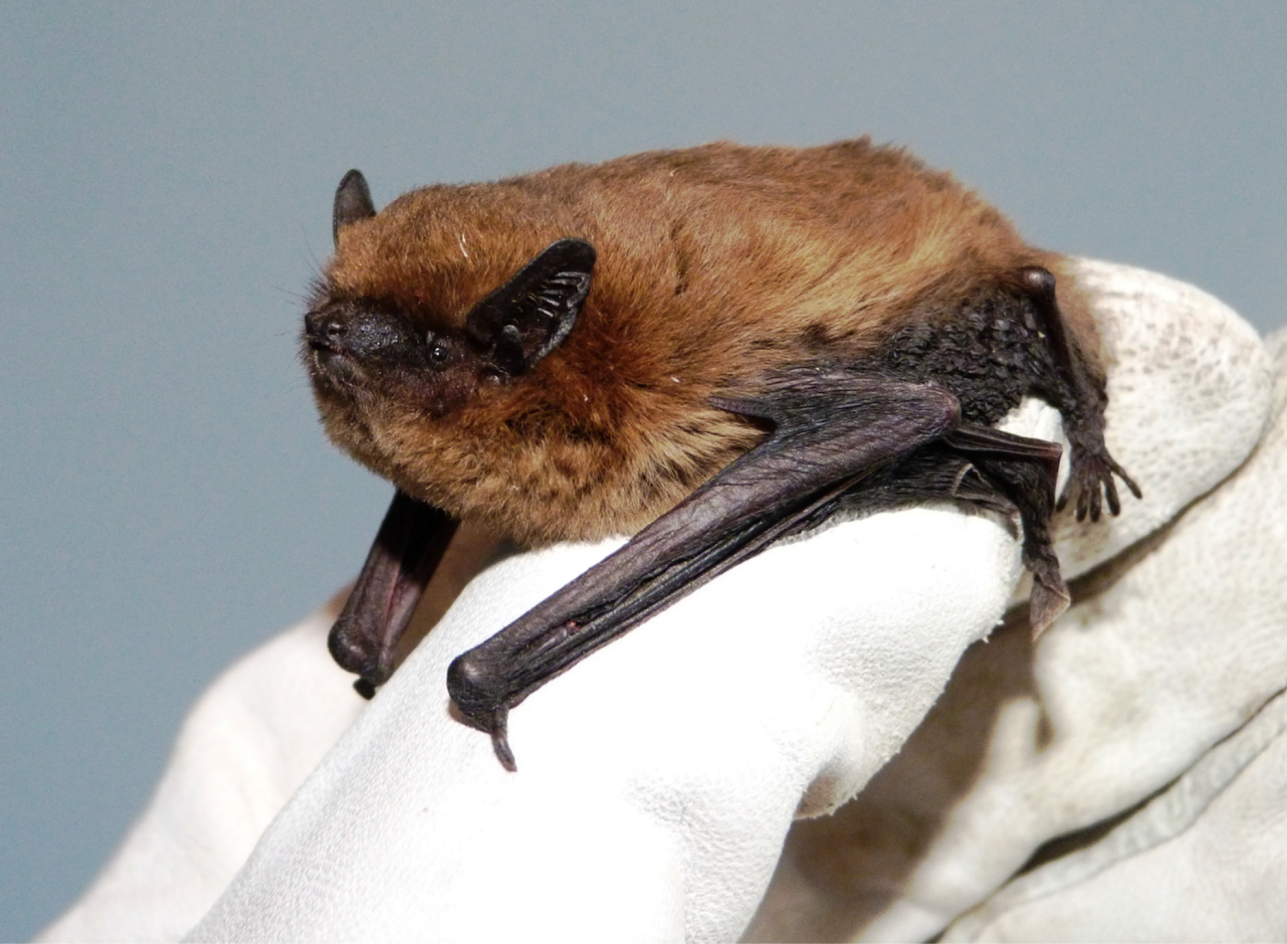
Bats in rehabilitation, such as this young common pipistrelle, could potentially be infected by SARS‐CoV‐2 if their rehabilitators have been in contact with the virus; hence, bat testing needs to be in place before safe release into the wild (photo: Daniel Hargreaves/bat conservation trust).

Samples have been successfully collected and tested for three common pipistrelles (*Pipistrellus pipistrellus*; Figure 1) following contact with individuals confirmed SARs‐CoV‐2 positive. Of these, the first bat was found in the home of a family self‐isolating following positive SARS‐CoV‐2 testing of a child within the home. Although the bat did not have direct contact with the child, proximity to this individual and contact with those self‐isolating within the house warranted SARS‐CoV‐2 testing before consideration for release back into the wild following rehabilitation. The other two animals were a pair raised in captivity, and their carer tested positive for SARS‐CoV‐2. As described above, three fecal samples were provided per animal, tested, and all confirmed negative by RT‐qPCR. In addition to these bats, the RNA extraction and RT‐qPCR protocols described have been implemented as part of a larger project investigating the prevalence of SARSr‐CoV in UK bats by Imperial College London. In this project, more than 250 bats have been screened, with several animals being identified to be carrying SARSr‐CoV but not SARS‐CoV‐2 (unpubl.).

## 4 CONCLUSION

The reporting of SARS‐CoV‐2 infection in wild and domesticated animals is a requirement under both UK legislation (The Zoonoses Order) and under our membership as part of the World Organization for Animal Health (UK Government, 2021b). This strategy is to allow government to fulfill national and international reporting obligations and to improve the evidence base regarding the potential role of animals during the current and future pandemics. Such a protocol is readily adaptable and could additionally be purposed for such roles as population sampling in communal roosts as part of future biomonitoring of wild bats. There is a growing need to understand the potential for wildlife to act as reservoirs, as well as the risk of human to wildlife transmission, particularly to avoid any inappropriate requests for formal disease control measures as well as to discourage misinformed illegal eradication undertaken by citizens, and still considering the public health risk. The present protocol has been designed with the best interests of both the wildlife and their rehabilitators in mind: developing noninvasive strategies and working closely with policymakers and disease experts is a good example of how the emerging call for “One Health” approaches, which consider both humans and wildlife together can be put into practice.

## Data Availability

There is no data associated with the aper but PCR results (output from qPCR machine) are available from the first and/or the corresponding author.
